# Optimized Machine Learning for the Early Detection of Polycystic Ovary Syndrome in Women

**DOI:** 10.3390/s25041166

**Published:** 2025-02-14

**Authors:** Bharti Panjwani, Jyoti Yadav, Vijay Mohan, Neha Agarwal, Saurabh Agarwal

**Affiliations:** 1 Department of Computer Science & Engineering, Shri Madhwa Vadiraja Institute of Technology and Management, Bantakal 574115, Karnataka, India; bharti.cs@sode-edu.in; 2 Department of Instrumentation and Control Engineering, Netaji Subhas University of Technology, Sector-3 Dwarka, New Delhi 110078, Delhi, India; jyoti.yadav@nsut.ac.in; 3 Department of Mechatronics, Manipal Institute of Technology, Manipal Academy of Higher Education, Manipal 576104, Karnataka, India; 4 School of Chemical Engineering, Yeungnam University, Gyeongsan 38541, Republic of Korea; 5 Department of Information and Communication Engineering, Yeungnam University, Gyeongsan 38541, Republic of Korea

**Keywords:** polycystic ovary syndrome detection, ensemble learning, deep learning, walrus optimization algorithm, cuckoo search algorithm

## Abstract

Polycystic ovary syndrome (PCOS) is a medical condition that impacts millions of women worldwide; however, due to a lack of public awareness, as well as the expensive testing involved in the identification of PCOS, 70% of cases go undiagnosed. Therefore, the primary objective of this study is to design an expert machine learning (ML) model for the early diagnosis of PCOS based on initial symptoms and health indicators; two datasets were amalgamated and preprocessed to accomplish this goal, resulting in a new symptomatic dataset with 12 attributes. An ensemble learning (EL) model, with seven base classifiers, and a deep learning (DL) model, as the meta-level classifier, are proposed. The hyperparameters of the EL model were optimized through the nature-inspired walrus optimization (WaO), cuckoo search optimization (CSO), and random search optimization (RSO) algorithms, leading to the WaOEL, CSOEL, and RSOEL models, respectively. The results obtained prove the supremacy of the designed WaOEL model over the other models, with a PCOS prediction accuracy of 92.8% and an area under the receiver operating characteristic curve (AUC) of 0.93; moreover, feature importance analysis, presented with random forest (RF) and Shapley additive values (SHAP) for positive PCOS predictions, highlights crucial clinical insights and the need for early intervention. Our findings suggest that patients with features related to obesity and high cholesterol are more likely to be diagnosed as PCOS positive. Most importantly, it is inferred from this study that early PCOS identification without expensive tests is possible with the proposed WaOEL, which helps clinicians and patients make better informed decisions, identify comorbidities, and reduce the harmful long-term effects of PCOS.

## 1. Introduction

PCOS is a complex, multi-faceted ailment found in women of reproductive age; it is a commonly occurring endocrine disorder in women that results in hormonal imbalance, high androgen levels, irregular periods, and infertility. It is estimated that up to 8–13% of women worldwide are affected by PCOS during their reproductive period [1]. Studies also suggest that, with lifestyle changes and unbalanced diets, the prevalence of PCOS has increased in the recent past [2]; for example, according to a cross-sectional study conducted in India, about 21.13% of adolescent girls in their teenage years are affected by PCOS, yet this is likely to be an underestimation [3]. PCOS affects the person both biologically and psychologically, leading to mental health barriers and social ignominy.

PCOS is characterized by excess amounts of androgen, oligo-anovulation, and polycystic ovaries, resulting in hormonal imbalances; the cysts circumspectly inflate, potentially blocking the ovulation process and decreasing the chance of becoming gravid, thereby causing infertility [4]. PCOS leads to a complex metabolic syndrome associated with long-term health risks, including insulin resistance, altered fibrinolytic activity, and dyslipidemia. The literature reveals that women with PCOS have higher risks of developing uterine cancer, type 2 diabetes, and blood vessel diseases [5,6,7], as the secretions of metabolic hormones, such as leptin and adiponectin, continually fluctuate, contributing to metabolic derangements [8]. The biochemical manifestations of PCOS further hamper health efforts, augmenting obesity and weight gain [9,10]. Some researchers say that insulin resistance (IR) is one of the key elements causing increased androgen levels and reduced oocyte quality; evidence from large cohorts suggests that IR and β cell dysfunction promote the progression to impaired glucose homeostasis, resulting in a high probability of dysglycemic conditions in patients with PCOS [11,12]. The authors in [13,14] revealed that women affected by PCOS have a 40% higher chance of developing diabetes and obesity; furthermore, according to a systematic study conducted recently, patients with PCOS had higher HDL serum concentrations and were at higher risk of hypertension, as well as cerebrovascular disease, compared to women without PCOS [15,16], with studies suggesting that PCOS exacerbates heart health conditions and increases the risk of stroke by almost 50% [17,18].

World Health Organization (WHO) statistics report PCOS as the most common cause of infertility; government authorities also identify PCOS as a severe health issue in women that not only causes anxiety and depression, but may also lead to obesity, systemic inflammation, hypertension, cardiovascular disease (CVD), endometrial cancer, and other metabolic disorders, if not treated properly. The socio-economic burden in the management of PCOS-related symptoms and morbidity is in the billions of USD annually [19]. Furthermore, due to lifestyle changes, the number of cases of PCOS per year is increasing drastically; for the well-being of women across the globe, there is a need for strategic planning, proper monitoring, and enhancement of public awareness around PCOS [20,21]. 

The early detection of PCOS can help in better management of emotional and physiological symptoms, while potentially avoiding critical ailments altogether; however, according to a report from the WHO, up to 70% of PCOS cases worldwide go undiagnosed [22,23]. The symptoms presented in PCOS differ from person to person and occur without any apparent trigger; the clinical diagnosis of PCOS is based on Rotterdam criteria, characterized by oligo-anovulation, hyperandrogenism, and polycystic ovaries, but the condition is a complex, heterogeneous phenotype, affected by heredity and intra- or extra-uterine pathophysiological conditions. Therefore, the criteria-wise diagnosis of PCOS presents a challenge to clinicians, a complication that is compounded due to variations in the assessment of menstrual irregularity, environmental factors, oxidative stress, and genetic factors [24,25]. Unfortunately, less than 50% of women are appropriately diagnosed and receive proper medical care leading to improved management of symptoms and lifestyle [26,27,28].

Bioinformatics and artificial intelligence (AI) techniques developed over the past decade have revolutionized the process of disease diagnosis and could play instrumental roles in medical management [29,30,31,32]. Intelligent systems, namely ML algorithms, DL networks, integrated learning, and metaheuristic algorithms, include established models that can analyze medical data and identify diseases precisely and reliably [33,34,35,36,37]. Various learning algorithms, such as the K-nearest neighbor (KNN), support vector machine (SVM), logistic regression (LR), decision tree (DT), and random forest (RF) algorithms, among others, are fairly prevalent in the domains of prediction and classification [38,39]; in addition, these AI models can learn continuously from current observations and history, and therefore have the ability to provide accurate intervention information [40,41]. Table 1 documents the comprehensive use of AI systems to detect PCOS, cardiovascular ailments, diabetes, and other prevalent medical conditions.

It can be inferred from the detailed literature survey that data-driven intelligent models have the potential to offer clinicians vital information with which to achieve enhanced early diagnosis, thereby offering better and more effective patient care [50,51,52]. Some recent relevant work related to research in the PCOS domain includes reference [53], in which a proposed fuzzy data transformation model was used with hormonal data to diagnose a broader spectrum of PCOS, as the ML models presented the output in three categories instead of two. The authors of [54] proposed the AdaBoost model for PCOS prediction while utilizing different feature selection techniques, namely the standard Scaler and mutual information techniques. The major limitation identified in the current research is that it provides PCOS prediction using a dataset of symptoms and test results from invasive tests and ultrasound, an issue that Zigarelli et al. [55] tried to address, presenting a self-diagnostic platform with an RF model that considers symptomatic and clinical test data subgroups. The model performs satisfactorily, with an 11% improvement in prediction with the sub-division of data; however, the accuracy achieved could be further improved using expert models. Aggarwal et al. [56] proposed feature selection and data amalgamation for datasets to find the relationships between lifestyle diseases and PCOS. The study included seven different supervised and unsupervised learning methods, with gradient boost classification exhibiting the most accurate prediction; however, the authors of the study assumed that PCOS is 100% associated with diabetes and considered insulin resistance as the target variable, which is not accurate for 100 percent of PCOS cases. Therefore, the prime objective of this work was to leverage the available system intelligence for the early symptomatic detection of PCOS in women.

As previously discussed, the majority of PCOS cases go undiagnosed in preliminary stages, owing to the condition’s intricate nature and the expensive tests involved; consequently, a significant number of patients with PCOS suffer from metabolic disorders such as diabetes, poor heart health, and hypertension in later stages [57]. Usually, women do not even undergo PCOS detection tests due to a lack of awareness, whereas standard tests, including fasting glucose tests and pulse and pressure measurements, are performed routinely; therefore, the idea behind this research is to predict the presence of PCOS based on the information obtained from primary symptoms and the general health indicators associated with lifestyle diseases. In order to attain our goal, the data from CVD and PCOS disease datasets were blended to obtain a new dataset, and then feature extraction and refining were conducted to identify 12 critical attributes exhibited by women with hormonal imbalances. It is important to note here that only relevant features related to obesity, diabetes, hypertension, and cardiovascular tests were considered, while other invasive hormonal test parameters were specifically discarded. The blended dataset was then used to train ML and DL models for the intelligent prediction of PCOS.

Predictive model development for medical purposes is a critical task, requiring high precision and accuracy; therefore, an ensembled technique with seven base learners, as well as a DL network for a meta-classifier, is proposed. The efficiency of the proposed EL models is highly dependent on the optimal values of the hyperparameters, whose retrieval requires eminent computational intelligence [58]. Consequently, a state-of-the-art metaheuristic WaO was employed to realize the optimal EL model (WaOEL) for the accurate prediction of PCOS. Furthermore, relevant studies suggest various algorithms for the hyperparameter estimation of ML models [59,60,61,62,63,64]; therefore, for comparison, conventional RSO and CSO models were also applied to evaluate the parameters of the designed EL model, resulting in RSOEL and CSOEL, respectively. The predictive performance of all of the models was thoroughly investigated for the blended symptomatic dataset. Furthermore, in order to provide clinical validation and transparency to the outcome of the designed models, feature importance analysis was carried out, utilizing RF and SHAP architecture, in order to assign ranks and values to contributing features for making the prediction based on a game theory approach [65]. Our analysis provides medical insights and establishes the relationships between features and positive PCOS prediction.

In the framework of this research, we attempt to predict PCOS from the symptoms women present in the early stages, rather than considering the complex attributes of dedicated hormonal tests concentrated on predicting the condition directly. To the best of our knowledge, this work is a maiden attempt to design an optimized ensembled technique-based learning model, namely WaOEL, for symptomatic early identification of PCOS. The smart data-driven intervention with the WaOEL framework provides patients with an opportunity to assess their susceptibility to PCOS with only basic symptoms; this early diagnosis of PCOS may help patients adopt a better lifestyle and take other preventive actions in order to moderate the progression of comorbid diseases. The primary contributions of this research are as follows:

- Through the amalgamation of two different datasets, an entirely new symptomatic PCOS dataset, with initial symptoms and fundamental health indicators, was created.
- Learning algorithms, such as KNN, LR, SVM, DT, RF, XGB, and DL, tuned with RSO, were implemented to identify PCOS. The efficiency of the designed models was evaluated and validated based on accuracy, precision, F1 score, cross-validation comparisons, and other metrics.
- Ensembled models were created with stacking techniques to further improve their performance; comparative analysis for different meta-level classifiers was conducted to identify the best-performing model.
- A metaheuristic WaO algorithm was proposed to design the optimally tuned EL classifier (WaOEL), with the DL network as the meta-classifier for symptomatic PCOS prediction.
- Other optimization techniques, including RSO and CSO, were also employed to design EL models. A rigorous performance comparison of the proposed WaOEL was made with CSOEL and RSOEL based on the convergence plot, fitness value, and prediction metrics.
- Feature importance analysis was carried out using the SHAP framework to interpret the predictions obtained using the designed models.

The subsequent sections of this article are organized as follows: Section 2 details the overview of the proposed framework of this study. Section 3 concisely summarizes the symptomatic PCOS dataset utilized, as well as the learning algorithms implemented, to realize the proposed predictive models; it also provides a brief description of the WaO algorithm. Section 4 presents an account of the outcomes of designed learning techniques with comparative analysis, further discussion of which is presented in Section 5. Finally, Section 6 presents the conclusions of this study, offering insights into early PCOS prediction.

## 2. Proposed Framework

**Figure 1:**
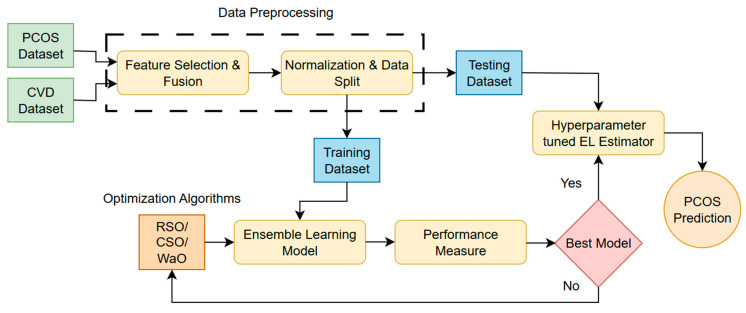
Schematic representation of the proposed framework.

The proposed framework for the symptom-based early identification of PCOS using an optimized EL model is illustrated in Figure 1. Initially, we obtained the datasets from Kaggle and performed preprocessing; during the pre-processing phase, male data samples were removed, and crucial features were identified and blended to obtain the symptomatic dataset. Thereafter, the text data underwent encoding, and the MinMax scaling approach was used for normalization. In the following stage, the data were partitioned into two categories, the training and testing datasets, with a ratio of 80:20. Diverse ML techniques, such as LR, KNN, SVM, DT, RF, XGB, and DL networks, were implemented, with hyperparameters tuned using RSO to predict PCOS through fitting with the symptomatic training dataset.

Following the above stage, the individual models were integrated using the stacking method to develop the ensembled models, thereby providing the ability to improve model generalization, reduce overfitting, and successfully handle complex data. All of the classifiers were used as base classifiers and trained on the training dataset with 10-fold cross-validation to make predictions. Subsequently, the prediction from the previous stage was input to a meta-learner to obtain the final prediction; however, the choice of meta-learner and the parameter values for each learner are crucial for accurate decision making. Consequently, when selecting the final classifier, all seven learning models were considered meta-learners, and their prediction accuracies were compared; the EL model with a DL network exhibited the best accuracy and was considered for further optimization.

Subsequently, parameter estimation for the ensembled learner was formulated as an optimization problem to solve, using a nature-inspired WaO algorithm developed in 2023. The WaO algorithm optimizes a total of 25 hyperparameters to maximize prediction accuracy. The model was trained with 80% training data, as well as 10-fold cross-validation, and examined for all possible hyperparameter combinations. Finally, this optimized expert WaOEL, with its fine-tuned parameters, was tested using the 20% reserved PCOS dataset. The aforementioned approach allowed robust hyperparameter tuning, while preserving a separate test set for the final assessment; the ensembled models were also optimized with RSO and CSO, with similar search ranges and the same objective to perform a fair comparison. Finally, the predictions of the ensembled models were analyzed using the unified framework, SHAP, to identify the important features and derive clinical inference. With this study, we deliver an optimal deep EL model, WaOEL, for early symptomatic PCOS identification without involving expensive, invasive tests.

## 3. Materials and Methods

### 3.1. Dataset Description

**Figure 2:**
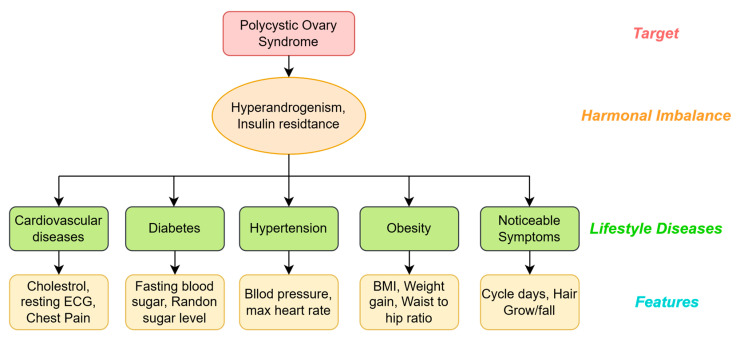
A grid showing the relationship between PCOS and its features.

The existing literature indicates that PCOS leads to hormonal imbalance, resulting in insulin resistance and hyperandrogenism; it is a physiological response of the body to insulin, preventing the absorption of sugar by cells and leading to elevated blood glucose levels related to diabetes. The disturbed hormones further cause increased testosterone levels in the bloodstream, a condition known as hyperandrogenemia, which is an essential symptom for PCOS diagnostic testing [66]; however, while women typically undergo routine screenings for well-known lifestyle disorders, they often lack knowledge about PCOS. The literature also suggests strong correlations between PCOS in women and the presence of heart disease, obesity, hypertension, or diabetes in later stages of life; therefore, we aim herein to envisage PCOS at an early stage from the biomarkers related to well-known lifestyle disorders, thereby avoiding dedicated invasive tests. Figure 2 illustrates the relationships among PCOS, the hormonal imbalances involved, and various disorders, highlighting the features related to them.

**Figure 3:**
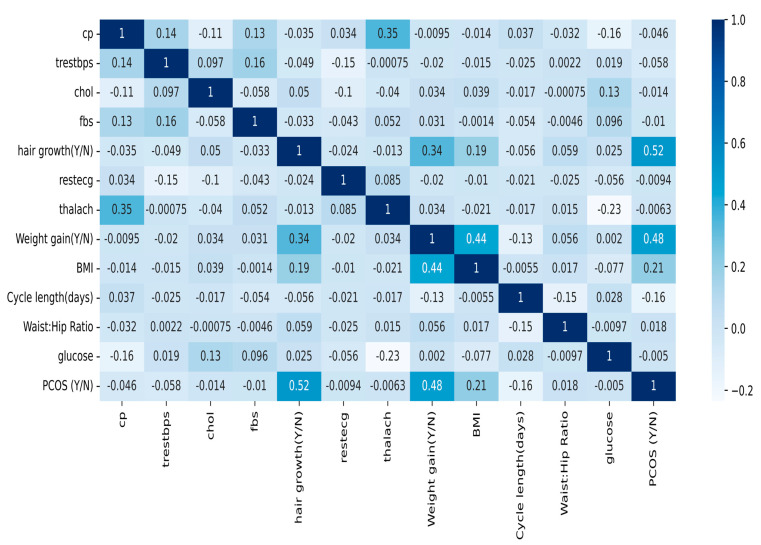
The correlation matrix between input and output attributes.

**Figure 4:**
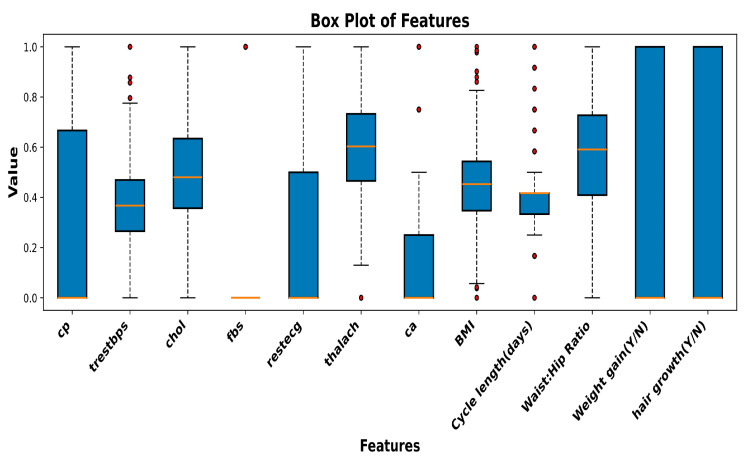
Boxplot analysis of the features.

**Figure 5:**
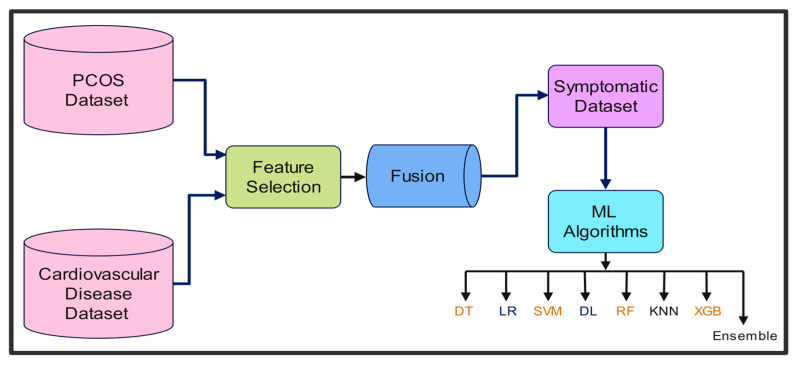
The steps to obtain the blended symptomatic dataset.

Based on the above principle, one of the aims of this investigation was to characterize a symptomatic dataset for the early prediction of PCOS that offers comprehensive coverage for lifestyle diseases, is readily accessible, consists of a limited number of features, and is computationally efficient; the following two datasets were used for these purposes: the CVD dataset [67] and the PCOS dataset [68]. Both datasets were taken from the authentic Kaggle repository, maintained by data scientists and engineers. Both individual datasets were preprocessed by initially examining for duplicate data and deleting the affected rows; thereafter, the dataset was examined for any missing or null values, and affected rows were filled with 0 for numerical data and the most occurring variable in the case of categorical data. Further processing was conducted after data amalgamation on the blended symptomatic PCOS dataset, the highlights of which are detailed below:

- Initially, feature selection was performed, and relevant attributes from various biomarkers and basic clinical tests were extracted from the raw datasets. The attributes related to hypertension, diabetes, obesity, and cardiometabolic disease were considered, while the attributes obtained from complex clinical tests were disregarded.
- The CVD dataset contains 1026 sample records for 13 attributes: age, sex, resting blood pressure, chest pain type, cholesterol, colored vessels in fluoroscopy, resting electrocardiogram, fasting blood sugar, maximum heart rate, angina, ST segment, thalassemia, and the target value. The recorded samples considered for further analysis included only women of reproductive age. The attributes selected for further analysis were cholesterol, fasting blood sugar, resting blood pressure, maximum heart rate, resting ECG, and chest pain.
- The PCOS dataset contains 44 parameters and 542 records, such as patient number, including age, weight, height, BMI, blood type, pulse rate, cycle length, marital status, random glucose, pregnancy, number of abortions, I beta-HCG, II beta-HCG, FSH, LH, FSH/LH, waist-to-hip ratio, TSH, AMH, PRL, vit D3, PRG, RBS, weight gain, hair growth, skin darkening, pimples, follicle number, average follicle size, endometrium, target PCOS, and a few others. The features determined using specific tests were removed, and only the basic noticeable parameters, such as BMI, glucose, cycle length, waist-to-hip ratio, weight gain, hair growth, and PCOS, were considered for further analysis.
- The new symptomatic dataset, after blending relevant symptoms, contains a total of 932 samples with 13 attributes, namely resting blood pressure (trestbps), maximum heart rate (thalach), cholesterol (chol), chest pain (cp), resting electrocardiogram (restecg), fasting blood sugar (fbs), random glucose (glucose), body mass index (BMI), weight gain, waist-to-hip ratio, cycle length, hair growth, and the result PCOS (Y/N). The dataset samples are shown in Table 2.
- The blended dataset contains both numerical and categorical data types and records a woman’s health parameters related to hypertension, heart health, obesity, diabetes, and noticeable hormonal effects. PCOS, being the target parameter, represents the patient’s PCOS diagnosis in relation to the attributes mentioned above. A null value denotes that the patient is not diagnosed with PCOS, and a value of one denotes that the patient suffers from PCOS.
- The obtained dataset is suitable for exploratory data analysis (EDA) to understand the relationships among various health indicators, potential biomarkers, and the outcome of PCOS. Figure 3 shows the correlation matrix between various input features and the target output. Negative coefficient values denote adverse relationships between input and output, i.e., an increase in one value results in a decrease in the other and vice versa.
- To develop an expert data-driven optimized model, boxplot analysis was used to examine the data for outliers. Figure 4 shows that most of the features have even distributions with clear medians; however, features such as waist-to-hip ratio, weight gain (Y/N), and cycle length (days) have several outliers. The outliers in the numerical attributes of the dataset were trimmed, resulting in 900 samples with 12 features.
- Data normalization was carried out using the MinMax scalar function for feature scaling. Data normalization helped achieve promising results compared to the raw data, bringing uniformity to the data and advancing interoperability. The steps to obtain the blended symptomatic dataset are depicted in Figure 5.
- In order to evaluate the performance of the designed models, the dataset was split into training and testing datasets, with a ratio of 80:20.

### 3.2. Learning Models

ML and DL are the techniques primarily employed in the development of data-driven expert predictive models; the core concept is to analyze large chunks of data and understand the underlying intricate relationships to generate a mapping function, in a process known as training, which enables the trained models to predict the target value (Y) for new or unseen input features (X). Various ML models were developed in this work, namely LR, KNN, SVM, DT, RF, and XGB. The field of DL employs neural networks to learn the relationships between input features and target values. A DL model is a black box of neuron layers that adjusts the interconnecting weights according to the relationship between input features (X) and target value (Y).

In this work, we utilized RSO-tuned ML and DL learning models to classify patients at risk of PCOS based on only commonly known symptoms and health attributes; by combining the individual models, a stacked EL model was also designed. The performance of the models depends upon how well-tuned design variables are selected; therefore, the subsequent paragraphs provide a concise description of the models employed and their hyperparameters selected for tuning in this project.

#### 3.2.1. K-Nearest Neighbor

The KNN algorithm is a simple ML technique based on supervised learning, primarily used to handle classification problems [69]; it is a non-parametric algorithm that makes no assumptions based on the provided input data and is called a lazy learner algorithm because, in the training phase, it stores the data without classifying them. The algorithm compares the new data points with a group of comparable categories, then uses the Euclidean distance function described in Equation (1) to handle the data and classify the new data points. Here, $x_{1},\;x_{2},\;y_{1},\;and\;y_{2}$ are the arbitrary points of input data coordinates. Choosing the correct value of neighbor ‘k’ is a difficult task and can be achieved by analyzing the problem or optimizing it with a suitable technique.

(1) $$Euclidean\;Distance=\sqrt{{\left(y_{2}-y_{1}\right)}^{2}+{\left(x_{2}-x_{1}\right)}^{2}}$$

#### 3.2.2. Logistic Regression

One of the most well-known algorithms in supervised learning is LR, which provides binary predictions about categorical independent variables. LR employs the sigmoid function to generate predictive probabilities between 0 and 1, then estimates the likelihood of specific outcomes for new data with continuous or discrete values [70]. The final expression for the LR classifier is provided in Equation (2). For binary class LR, the cost function is defined with weights for the training sample, the probability of outcomes, and a regularization term. The objective considered for minimization is provided in Equation (3).

(2) $$log\left[\frac{y}{1-y}\right]=w_{0}+w_{1}X_{1}+w_{2}X_{2}+w_{3}X_{3}+\cdots +w_{n}X_{n}$$

(3) $$Obj1=\underset{w}{\min}\frac{1}{S}\sum_{i=1}^{n}s_{i}(-y_{i}\log\left(\hat{p}\left(X_{i}\right)\right)-(1-y_{i})\log\left(1-\hat{p}\left(X_{i}\right))\right)+\frac{r\left(w\right)}{SC}$$

Here, $\hat{p}\left(X_{i}\right)$ is the prediction probability, $s_{i}$ corresponds to the weights assigned for the *i*th sample, and *S* is the sum of all weights. *r*(*w*) is the regularization term for the LR coefficients, which helps to improve numerical stability in learning. The parameter chosen for optimization in implementing LR classification is the inverse of regularization strength, ‘*C*’, which helps in avoiding generalization error.

#### 3.2.3. Support Vector Machine

SVM is a skilled, supervisory ML technique for classification jobs [71]; the algorithm aims to identify the most effective hyperplane in order to split data points from various categories in an N-dimensional space. Support vectors are crucial in this technique to attain the maximum margins between data points of various classes. The dimensions of the hyperplane are correlated with the number of input features. For higher dimensional non-linear datasets, kernel functions are employed to enable complex separation boundaries. In this work, radial basis function (RBF) kernels and sigmoid kernels were considered due to the multi-dimensionality and complex nature of the medical data; the regularization parameter used to control the learning was determined with the help of an optimization algorithm.

#### 3.2.4. Decision Tree

DTs are mostly used to address classification problems in a structured manner, with internal nodes representing dataset properties, branches representing decision rules, and leaf nodes representing outcomes [72]. The test or decision-making process is guided by the features of the dataset provided for training, starting at the root node. The algorithm progresses by comparing dataset attributes and splitting them into sub-nodes. While creating a DT, great consideration goes into selecting the characteristics for the root node and any additional sub-nodes. The Gini index and information gain are two important attribute selection measures (ASMs) that assist in identifying the best split for each tree node. The generated subgroups split recursively until they reach the leaf nodes; hence, the other important parameters considered for optimization are minimum samples to split, minimum samples per leaf, maximum depth, and maximum number of leaf nodes.

#### 3.2.5. Random Forest

The RF algorithm belongs to the EL method, wherein a number of DTs are combined using the bagging technique to provide predictions [73]. The steps to create and train the RF model are described below.

- The algorithm initiates by randomly choosing ‘*k*’ features from the total ‘*m*’ features. The root node is determined using the best-split technique for the selected ‘*k*’ features.
- Child nodes are created until a specified depth is reached.
- Through the selection of different subsets of features, ‘*n*’ randomly constructed trees are obtained, which collectively form the RF.
- The bootstrap aggregating (bagging) approach facilitates tree learning in the RF training process.
- Bagging is carried out by continually replacing a random sample with another sample from the training set and fitting trees using these samples.

The diversity in creating trees is maintained by the randomness included in the algorithm, which helps to reduce variance and overfitting and enhances generalization to new data [74]; however, as the RF algorithm has a large set of hyperparameters that require exhaustive tuning, applying an optimization technique is apt for this problem. The vital parameters, such as the number of trees in the forest, the maximum depth for each tree, the minimum number of samples required for splits at internal nodes, the minimum number of samples to create a leaf, and the parameter controlling the randomness during the bootstrapping of data, are all considered for optimization.

#### 3.2.6. Extreme Gradient Boost Algorithm

The XGB algorithm is a technique categorized under the boosting framework of EL, with decision trees as its foundational unit; the system incorporates regularization methods to improve the overall performance of the model. Initially, the base learners are used to predict the target value, and the model is linked to the residual prediction; the next step is to fit the residual prediction, and the combined model performs better, with reduced errors [75]. The individual tree sizes are considered with less depth, and the number of trees is increased for optimization; likewise, parameters such as the number of trees, the learning rate, the depth of the tree, and the number of iterations are optimally selected. XGB provides excellent computational efficiency, feature analyzing ability, and capability for handling missing data.

#### 3.2.7. Deep Learning Network

A DL model is a black box that autonomously learns the correlation between the input data and the desired output with experience. In this study, we utilized a multilayer feed-forward perceptron (MLP) model to construct a DL model, with an input layer containing nodes equivalent to the number of features in the dataset, four hidden layers, and an output layer with a single neuron [36,37]. The hidden layer has numerous neurons interconnected in a unidirectional way, with varying weights updated through training. The output generated by each neuron ‘*k*’ can be mathematically expressed using Equation (4).

(4) $$f\left(x\right)=\varphi \left(\sum_{k}w_{k}x_{k}+b\right)$$

Here, $x_{k}$ and $w_{k}$ are the input and the weight of the input, respectively, whereas b and φ represent the bias and the activation function used for the neuron. The logistic sigmoid function was utilized as the activation function for hidden layers. The network was trained using the gradient descent back-propagation algorithm to update $w_{k}$ and b for the log-loss function, formulated as shown in Equation (5).

(5) $$loss=\frac{-1}{N}\sum_{i=1}^{N}\left[y_{i}logp_{i}+{(1-y}_{i})log(1-p_{i})\right]$$

Here, *N* presents the number of input samples, and *p* presents the probability of prediction. The DL network is promptly tuned, optimizing the hyperparameters of the network, such as the number of nodes in hidden layers, iterations for training, and learning rate.

#### 3.2.8. Ensemble Learning

EL is an intensive ML technique incorporating multiple algorithms to enhance accuracy and decision making. Ensemble techniques provide more robust results by combining the predictions of various models tested on the same dataset, each reflecting a distinct aspect of the underlying patterns [76]. The proposed EL model makes use of various viewpoint techniques, including bagging, boosting, and stacking, to create a novel and efficient model for PCOS screening. The training dataset was initially bootstrapped to train the multiple models at the base level, parallelly processing the dataset; the base models considered here were LR, KNN, SVM, DT, RF, XGB, and DL. Subsequently, the predictions from the base classifiers were input to the next layer of the classifier, known as the meta-classifier; through sequential training, the meta-classifier compensated for the shortcomings of its disparate models, and the amassed final model continuously improved the predictions. The classifier at the meta-level was chosen using the empirical method, in which each model was considered at the meta-level, and detailed analysis was performed.

The proposed EL model provides additional advantages, including better generalization, less overfitting, and effective management of large/small complicated datasets, but at the cost of increased computational complexity [77]. Additionally, the EL models require supplementary computational resources and training time compared to individual models, and the ensemble’s success is also contingent on the diversity and competence of its constituent models. Therefore, hyperparameter tuning with a state-of-the-art metaheuristic WaO algorithm was employed to boost the performance of the proposed EL technique, as presented in the subsequent section.

### 3.3. Walrus Optimization (WaO) Algorithm

An efficient predictive model needs properly tuned parameters to perform its best; therefore, hyperparameter optimization becomes an indispensable practice for identifying the ideal model parameters that control the learning process. Optimizing algorithms determine a set of parameter values by minimizing a predefined loss function for the given independent data with repetitive training and testing [78].

The WaO is a recently developed metaheuristic algorithm inspired by the intelligent behavior of a herd of walruses [79,80]. The WaO algorithm’s design is based on considerate decisions made by walruses while foraging, migrating, fleeing, and fighting. Each walrus, $X_{i}$, in the herd is considered a potential solution to the optimization problem, with its position, $x_{ij},\;$specifying the decision variables in the predefined search space. Initially, the walrus population is initialized with a random location vector and its fitness value, ${F(X}_{i})$, is calculated based on the objective function. The population size, N, and the number of decision variables to optimize, m, are fixed at the start of the algorithm. The superiority of a walrus as a best member is evaluated by its fitness with respect to the defined objective function. The positions of all of the walruses are updated with each iteration according to their decisions, and the mathematical formulation of the behavior of walruses is considered in three stages to develop the algorithm, as described below.

Stage 1: Foraging

Each solution is guided toward the present best solution, representing the foraging behavior wherein each individual is under the guidance of the most potent walrus for a food search; it improves exploration capability by scanning large areas in a search space and relocating the candidate with respect to the current strongest member, as per Equation (6). The objective function is re-evaluated, and the walrus position is updated if better fitness is achieved, as modeled in Equation (7).

(6) $$x_{ij}^{s1}=x_{ij}+\sigma *\left({BW}_{j}-I_{ij}{.x}_{ij}\right)$$

(7) $$X_{i}=\left\{\begin{array}{c} X_{i}^{s1}\;F_{i}^{s1}<F_{i}\\ X_{i},\;else \end{array}\right.$$

Here, $X_{i}^{s1}$ is the new position of the *i*th walrus and $F_{i}^{s1}$ is the fitness value obtained from the objective function for the candidate in the first stage. Additionally, σ is the random position update coefficient; ${BW}_{j}$ is the current best solution; and $I_{ij}$ is the exploration factor, with a fixed value of 1 or 2, which could be randomly chosen for lesser or broader exploration of the search area. i∈(1,2,3,…,N) and j∈(1,2,3,…,m); this stage helps in the discovery of the original optimal solution area through the utilization of previous knowledge.

Stage 2: Migrating

The solution is moved toward a randomly chosen destination, i.e., the location of another random walrus, to include their strong social migratory behavior. The herd of walruses naturally migrates to different locations with large displacements, according to the changing weather conditions, for better survival; this migratory behavior, updating the location of the walrus, is modeled using Equations (8) and (9). If the new location of the walrus achieves a better objective function value, it replaces the previous location; thus, this stage helps discover new areas in search space and avoid local optima.

(8) $$x_{ij}^{s2}=\left\{\begin{array}{c} x_{ij}+\sigma *\left(x_{kj}-I_{ij}.x_{ij}\right),\;F_{k}<F_{i};\;\\ x_{ij}+\sigma *\left(x_{ij}-x_{kj}\right),\;else; \end{array}\right.$$

(9) $$X_{i}=\left\{\begin{array}{c} X_{i}^{s2},\;F_{i}^{s2}<F_{i};\\ X_{i},\;else; \end{array}\right.$$

Here, $X_{i}^{s2}$ is the new location of the *i*th walrus, and $F_{i}^{s2}$ is the fitness value obtained from the objective function for the candidate in the second, i.e., migratory, stage. Additionally, the selected walrus, toward which the *i*th walrus would migrate, is represented by $X_{k},\;for\;k=\left\{1,2,3,\ldots ,N\right\},\;where\;k\neq i$ and has the fitness value of $F_{k}$; this step is very important, as it prevents dependency on one particular solution and avoids early convergence of the algorithm.

Stage 3: Fleeing or fighting

Introducing small positional changes in the current solution to achieve a better location during the fight incorporates the strategic fight-or-flight decision-making capability shown by the walrus. To formulate this behavior, the radius of the considered solution is kept variable; with each iteration, the position of the walrus is updated in the local vicinity between the lower and upper bound, as specified by Equations (10)–(12), to achieve better fitness than the previous location.

(10) $$x_{ij}^{s3}=x_{ij}+\left[{lp}_{j}^{t}+\left({up}_{j}^{t}-\delta *{lp}_{j}^{t}\right)\right]$$

(11) local bounds : lpjt=lpjt,upjt=upjt,

(12) $$X_{i}=\left\{\begin{array}{c} X_{i}^{s3},\;F_{i}^{s3}<F_{i},\\ X_{i},\;else; \end{array}\right.$$

Here, $X_{i}^{s3}$ is the new location for the *i*th walrus, and $F_{i}^{s3}$ is the fitness value obtained in the third stage for the variable value $x_{ij}^{s3}$. The lower and upper positions for a particular *j*th variable are specified as ${lp}_{j}^{t}$ and ${up}_{j}^{t}$, respectively. δ is a random change coefficient used to update the location of the walrus within specified bounds. Modeling this behavior increases the WaO algorithm’s exploitation capability of the local area around the existing solutions.

After updating the locations of the walrus using the defined 3-stage analytical process, the first iteration is finished. The location of each walrus here specifies a set of particular design parameter values for the models, evaluated using Equations (6)–(10). For each set of design parameters specified by a walrus location, the corresponding objective function value, i.e., fitness value, is evaluated. The location of each walrus is updated with this three-stage decision strategy if better fitness is achieved, thereby providing a refined set of design parameters within specified bounds with each iteration; the process is repeated with updated locations and improved walrus fitness for a specified number of iterations or until the stopping criterion is met. The algorithm returns the walrus with maximum strength, i.e., the solution candidate with the best fitness value. The stochastic nature of this algorithm provides structured heuristic exploration for optimal solutions; at the same time, its arbitrary behavior assures exploitation and diversity in its solutions. Therefore, the robust and adaptive nature of the WaO approach is suitable to identify the best combination of hyperparameters for complex EL models with a large number of design variables.

**Figure 6:**
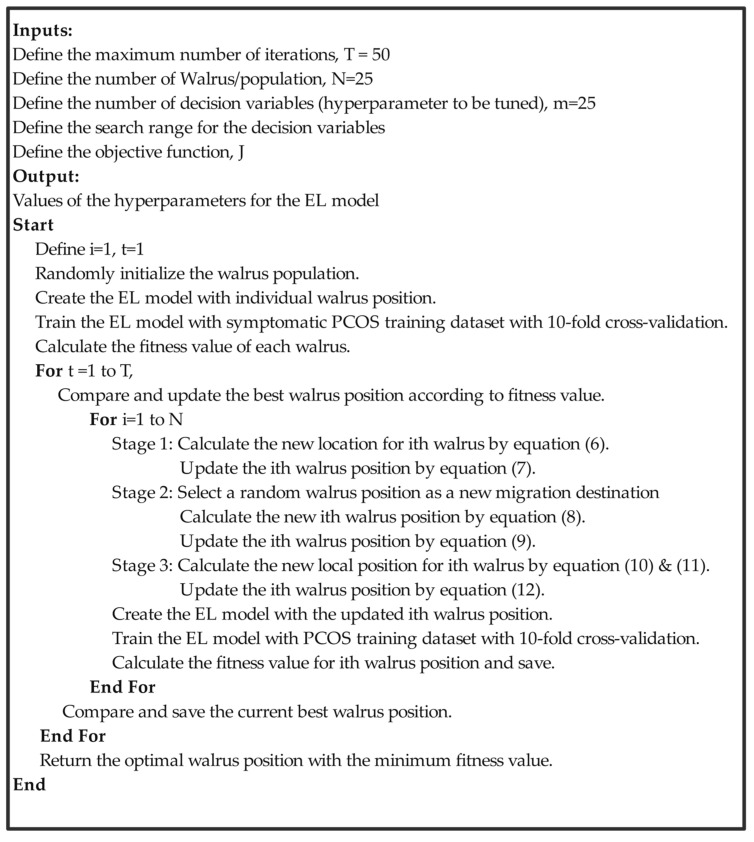
Implementation steps of WoA algorithm for EL model.

As the WaO algorithm is applied to obtain maximum efficiency, and only training data are to be considered for hyperparameter tuning, 10-fold mean cross-validation accuracy is the most suitable fitness function. Therefore, the objective function for minimizing was formulated as described in Equation (13); the WaO was implemented with the governing parameters described in Table 3 in order to minimize the defined objective function. The number of maximum iterations considered, 50, and population, 25, were key parameters that struck a perfect balance between exploration and exploitation. A large population or iteration may result in exploring a large number of solution candidates at the cost of increased time and computational complexity. The WaO algorithm is an excellent algorithm for such hyperparameter tuning, as it provides optimized solutions with a structured approach, but with very few decision variables; the steps for the implementation of the same are shown in Figure 6. It is important to note that RSO and CSO were also utilized for hyperparameter tuning to perform the comparative analysis; in order to make a fair comparison, the same ranges of parameter distribution, objective function, and population were considered for the RSO, CSO, and WaO tuning of the EL model.

(13) $$J=\frac{1}{Mean\;Crossvalidation\;Accuracy}$$

## 4. Results

### 4.1. Simulation Setup and Evaluation Metric

All of the proposed models presented in this work were implemented in Python’s Integrated Development and Learning Environment (IDLE Shell), version 3.11, 64-bit, on the Windows 11 platform using different data science and ML packages. In order to implement the advanced WaO algorithm, MATLAB R2023b was interfaced with Python to optimize the EL model. The system hardware configuration included 32 GB RAM, with a 13th Gen Intel(R) Core™ i9-13900K processor, and NVIDIA GeForce RTX 3070 with 8 GB GPU.

The models were built with a ratio of 80:20 split between training and test data for the blended symptomatic dataset. The training data were further divided 10-fold for cross-validation and hyper-parameter tuning. Learning curve and mean cross-validated accuracy were utilized to evaluate the robustness of the proposed models. 

The receiver operating characteristic (ROC) curve is a reliable method with which to evaluate binary classification capability in a graphical form. The AUC primarily specifies the degree of separability, where a higher AUC value specifies better overall performance of the classifier across various threshold states. The final trained models are examined for new unseen test data to assess their capabilities for the identification of PCOS. For binary classification, the confusion matrix works as an efficient tool with which to evaluate the model efficacy and provide a detailed report; therefore, the performance assessment for all of the developed models was conducted on metrics, such as accuracy, precision, sensitivity, specificity, F1 score, negative predictive value (NPV), false positive rate (FPR), and false negative rate (FNR), obtained from the confusion matrix.

### 4.2. PCOS Prediction Using Learning Models

This subsection presents a detailed analysis of the results obtained using designed optimized learning methodologies for the symptom-based prediction of PCOS. A comparative analysis of the performance of the individual models, namely, KNN, SVM, LR, DT, RF, XGB, and DL, with hyperparameters tuned using the RSO, was initially presented. The blended dataset, comprising 12 important features for the early diagnosis of PCOS, was split, with 80% of the data used as training data, while the models were tested using the remaining 20% of the unseen data. The mean cross-validated accuracy during the fitting of the model with the symptomatic training dataset is presented in Table 4; it is fair to acknowledge that all of the designed learning models performed satisfactorily.

**Figure 7:**
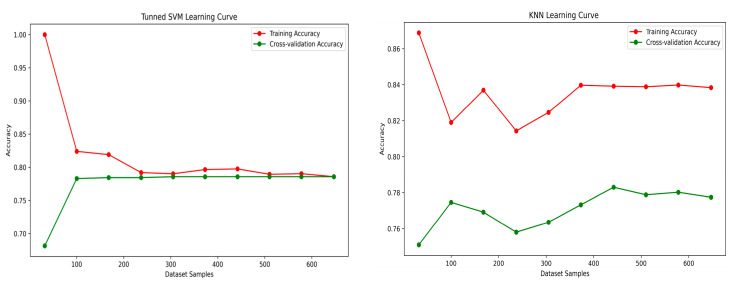

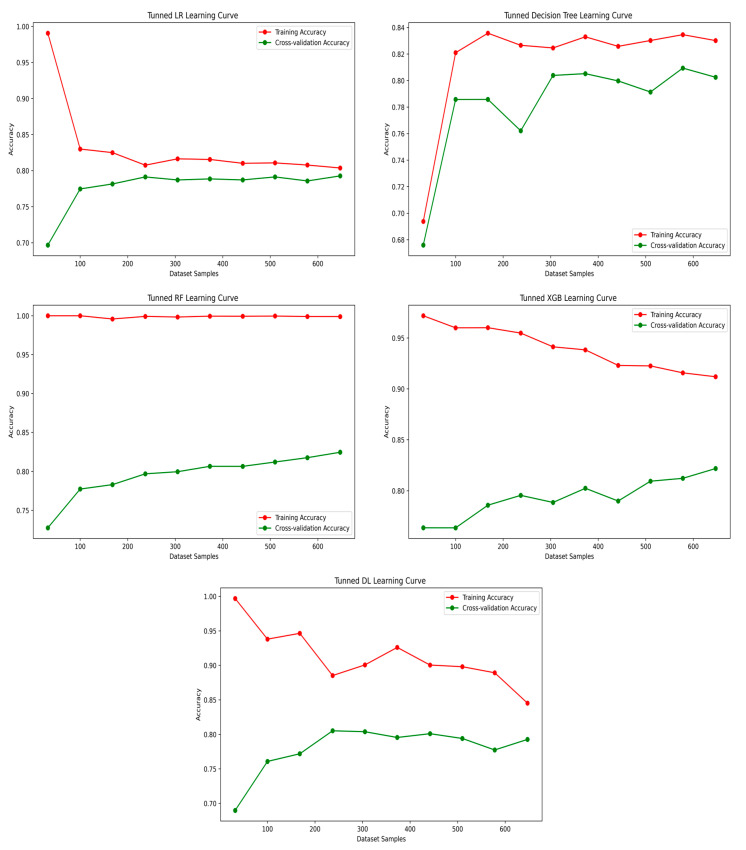
Learning curve for base models with the training dataset.

It is clear from the table that the RF classifier has the best mean cross-validation accuracy, 84.1%, proving its capability to generalize. In contrast, the KNN classifier has the relatively lowest accuracy, 79.3%. A learning curve was plotted with accuracy as a scoring metric versus training data samples to illustrate the predictive performance of the model with experience; it can be seen from Figure 7 that, initially, the accuracy in training was high, but it slowly reduced with increasing dataset size as the model tried to capture the association. Meanwhile, the cross-validation accuracy increased with the size of the dataset, suggesting an improvement in prediction accuracy with increased experience. It can be inferred that all of the learning models, except RF, demonstrated significant improvement with sample size; in contrast, it is observed, in the case of the XGB model, that learning could further improve with increased dataset size.

**Figure 8:**
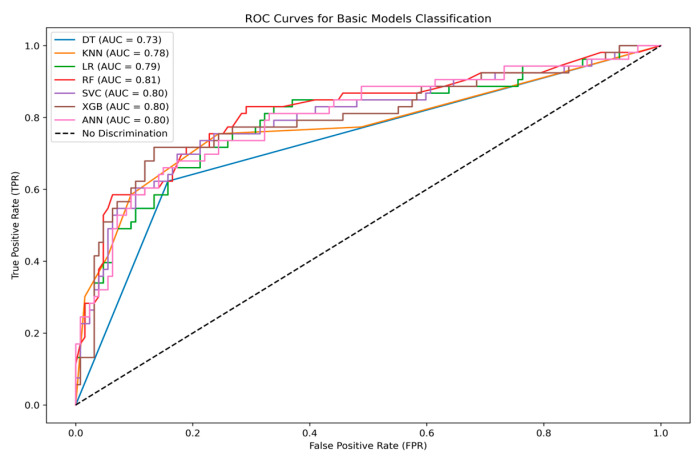
ROC comparison for base learning models.

Table 5 shows the performance of the ML and DL models with the entirely unseen testing data for all metrics for predicting PCOS. The RF classifier exhibited the highest classification accuracy and better precision than all other models; moreover, the ROC curve is shown in Figure 8, and the AUC value thoroughly evaluates the model’s classification efficacy. The RF classifier showed the most significant capacity to differentiate between positive and negative instances of PCOS, as demonstrated in its AUC value of 0.81.

### 4.3. PCOS Prediction with EL Model

Stacked EL models have proven to be efficient in different scenarios with complex computational problems; therefore, in this work, an ensemble technique with a stack of seven base classifiers and a meta-classifier was proposed. Various EL models were designed with different classifiers at the meta-level, tuned through the traditional RSO to attain optimal performance. The dataset was split, with an 80:20 ratio of training validation and testing set. The training data were further split 10-fold for hyperparameter tuning cross-validation, and the robustness of the models was examined using the remaining unseen testing data. The performance scores for predictions made using the testing data were calculated based on the confusion matrix and are listed in Table 6.

It is important to acknowledge that the KNN, XGB, and DL models performed better as meta-classifiers than other models, with 89% accuracy. Here, the DL classifier, at the meta-level, showed the best performance in accurately discriminating between positive and negative PCOS cases with the highest accuracy, precision, and specificity; therefore, for the design of the proposed optimal EL technique (WaOEL), the meta-classifier considered was the DL model.

### 4.4. Optimization of the EL Model

The ensemble technique and randomized search resulted in significant improvements in accuracy and other metric scores for symptomatic PCOS detection; therefore, a state-of-the-art intelligent algorithm should be utilized to augment predictive capability through optimal tuning of the EL model. However, as per the No Free Lunch (NFL) theorem, there is no particular algorithm that provides the best solution for all optimization problems [81]; thus, it is rational to employ multiple approaches in order to attain the quasi-optimal solution and choose the best solution. Consequently, along with the traditional RSO, a contemporary metaheuristic CSO, as well as present-day WaO algorithms, were employed for comparative analysis of the optimized EL predictive model. As far as the authors know, this is the first time that these algorithms have been compared.

**Figure 9:**
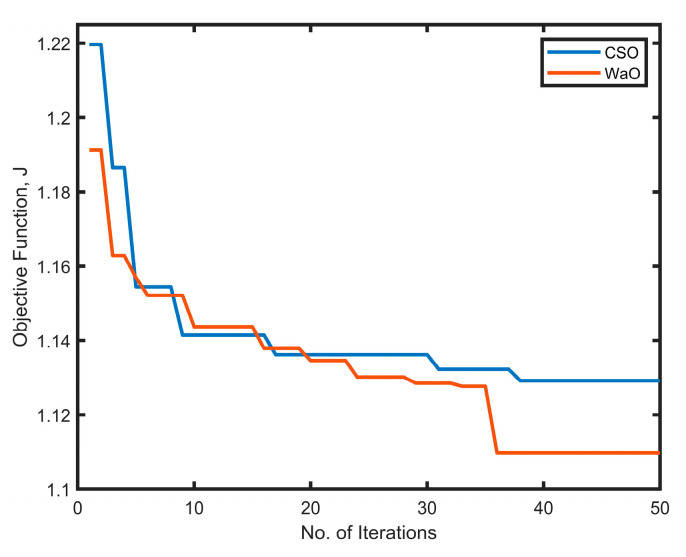
The convergence plot for CSO and WaO.

The vital hyperparameters of the learning models considered for optimization with the respective search space are outlined in Table 7. Figure 9 shows the convergence plot for WaO and CSO, and it can be seen that both of the metaheuristic algorithms converge fairly to generate optimal solutions; however, WaO achieves a minimum fitness value of 1.0778 within 35 iterations as compared to CSO, which reaches a fitness value of 1.091 after 45 iterations. The optimized hyperparameters for the ensembled model with various tuning methods are also presented in Table 7.

### 4.5. PCOS Prediction with Optimized EL Model

The EL models were then fitted with the symptomatic PCOS training dataset with the optimized hyperparameters obtained using the RSO, CSO, and WaO algorithms. Table 8 shows the average 10-fold cross-validation accuracy obtained during training; it can be seen that all of the optimized EL models perform better than individual models, with an almost 10% higher mean validation accuracy.

**Figure 10:**
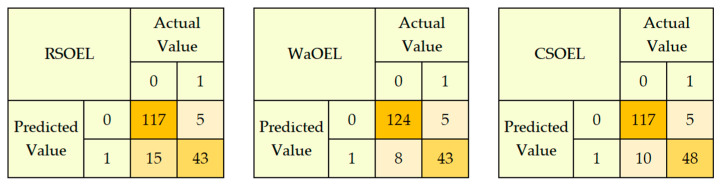
Confusion matrix of optimized EL models for PCOS detection.

The performances of the designed RSOEL, CSOEL, and WaOEL models for the detection of PCOS were also analyzed for completely unseen testing data. An extensive comparative analysis, based on the confusion matrix for prediction on 180 unseen data samples, is depicted in Figure 10.

The performance metrics evaluated from the confusion matrix for optimized EL models are presented in Table 9, where it can be seen that WaO achieved the highest accuracy, 92.8%, as compared to the 91.7% and 89.9% achieved using CSOEL and RSOEL, respectively. Here, sensitivity measures the capability of the designed model to correctly predict positive PCOS cases. It is worth noting that, quantitatively, the sensitivity of WaOEL is 25.1% higher than RSOEL and 1.2% better than CSOEL; however, the prediction for negative PCOS cases, represented by specificity, is higher for RSOEL by 3% in comparison to WaOEL. In the case of bioinformatics interventions for disease predictions, high precision is the primary required attribute. The WaOEL model achieved the highest precision, 92.7%, signifying the best identification of positive cases as compared to all of the other models. It can be inferred from the results that the WaOEL model exhibited the best performance, establishing its supremacy in the prediction of PCOS.

**Figure 11:**
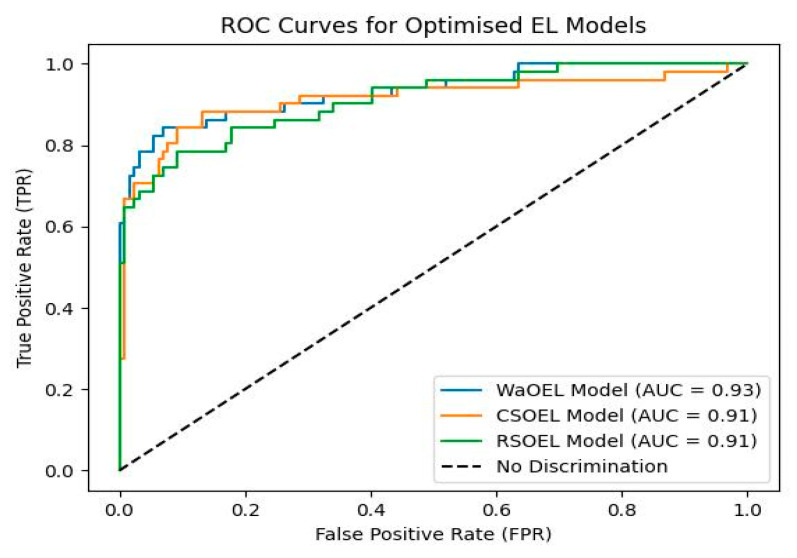
ROC comparison for optimized EL models.

The relationships between target PCOS and the symptomatic features are too complex to be learned in training with only 600 data samples; however, the advanced ensembled technique, with proper hyperparameter tuning using the WaO algorithm, enabled the models to learn complex associations. The ROC curve obtained for optimized models, depicted in Figure 11, proves the efficient predictions obtained with RSOEL, CSOEL, and WaOEL. The AUC value obtained for WaOEL is 0.93, as compared to 0.91, obtained with both CSOEL and RSOEL. It is noteworthy that all of the optimized EL models have quite high AUC values compared to 0.81, the highest obtained with individual RF models, again proving that the EL model with the DL network obtains the best prediction performance at the meta-level, optimized with the WaO algorithm.

**Figure 12:**
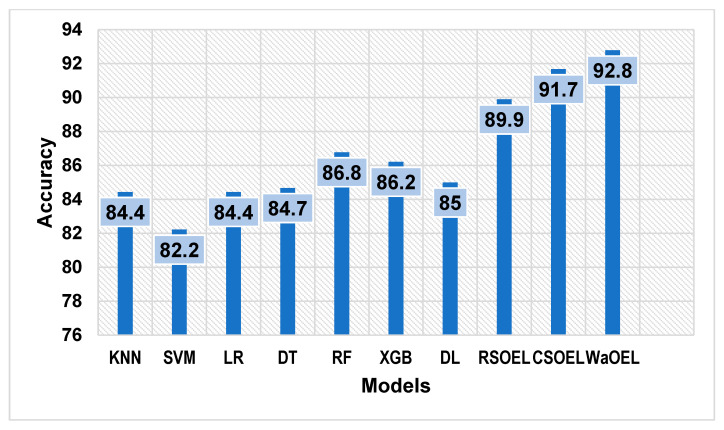
Accuracy comparison of different learning models.

The histogram depicted in Figure 12 illustrates that the ensembled optimal models consistently attain superior accuracy compared to their basic counterparts, which underscores the significance and efficacy of these strategies in enhancing the prognostic capability and overall efficiency of ML models, results implying that a neural network model, as a meta-level classifier, can considerably improve the prediction accuracy, especially when coupled with suitable hyperparameter adjustments with metaheuristic algorithms.

## 5. Discussions

In this section, we analyze the interpretation of the classifiers employed to provide predictions for symptomatic PCOS diagnosis. The designed WaOEL model is a multi-level stacking structure, comparable to other tree-based ensemble models; therefore, the RF and XGB ensembled models were considered for the feature importance and SHAP analyses. The acquired visualizations help to understand the feature dependence on the patient’s likelihood of being diagnosed with PCOS, thus adding clinical significance to the designed models.

- Feature importance with RF

**Figure 13:**
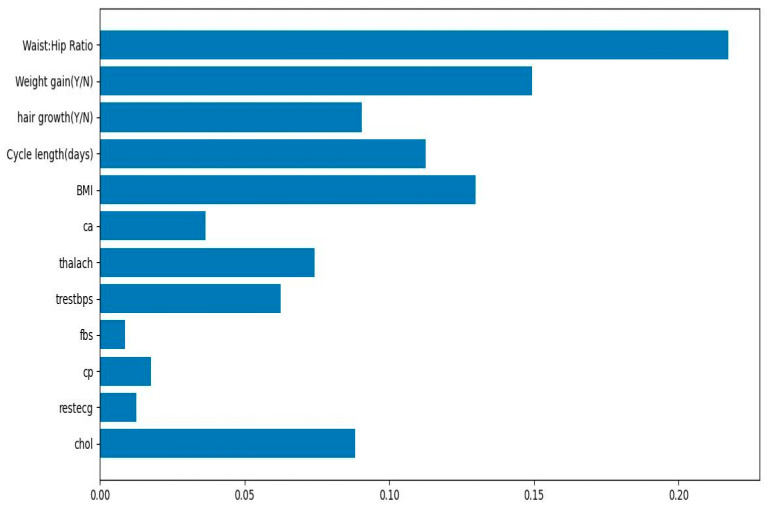
Feature importance with RF model.

The RF classifier is a very efficient tool for providing visualization and insights about the features considered for the PCOS predictions in this work. In order to determine the feature importance, we used Gini impurity to measure how well each feature decreased the impurity of the splits, a method that helps to determine the attribute that has the most influence on the model. Figure 13 is a graph of features ordered by their importance values; it can be seen from the graph that waist-to-hip ratio, weight gain, BMI, and cholesterol contributed significantly to a positive prediction of PCOS. It can therefore be inferred that sudden metabolic changes in the body may be due to PCOS as an underlying cause, and the model additionally suggests that patients with obesity are more likely to be diagnosed with PCOS, as supported by the medical literature [82]. Moreover, it can be seen that the proposed models interpret the basic symptoms just as practitioners would consider them during the primary detection of PCOS, and a positive PCOS prediction with higher importance attributed to features such as BMI and weight gain clinically signifies a metabolic phenotype generally characterized by insulin resistance and metabolic disorders, leading to diseases such as obesity. Hence, due to early intervention with the model, clinicians may adopt a more comprehensive approach to patient care and suggest weight management strategies or dietary changes.

b. Feature importance for XGB with SHAP

**Figure 14:**
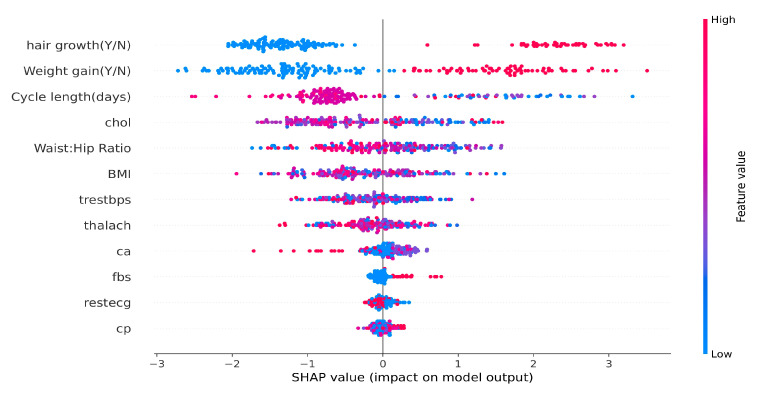
Feature importance with XGB model using SHAP.

Lundberg et al. [65] introduced the SHAP architecture, which assigns an important value to each attribute. SHAP measures the contributions of each feature that impact the model predictions, but it does not assess the quality of the outcome provided using the classifier [45]. The SHAP values were calculated for another ensemble structure, namely XGB, displayed in Figure 14.

The horizontal axis is for SHAP values of data points, with higher feature values shown in bright red and lesser feature values shown in blue. The best features, with higher impacts on decision making, are depicted at the top, with gradually lower importance until reaching the bottom level. The plot helps to identify the outliers and provides a more accurate representation of densities than kernel density estimates; it also reveals that sudden hair growth and weight gain are key factors in predicting PCOS. The prediction of PCOS patients using XGB includes factors such as cycle length, waist-to-hip ratio, BMI, and cholesterol, findings that align with the core clinical PCOS literature, in that excessive hair growth and weight gain with disturbed cycle lengths may be due to hormonal imbalances [83]. It is also important to note that patients with high cholesterol have higher chances of being diagnosed with PCOS. The model here signifies a very imperative underlying relationship between CVD and PCOS, due to their shared pathophysiology, with irregular lipid profiles and hormonal imbalances. Recognizing cholesterol as a primary contributing factor for PCOS using the proposed model might help clinicians conduct specific tests and devise personalized treatments in order to avoid long-term complications.

The WaOEL model designed in this work enables early symptomatic diagnosis of PCOS with only basic symptoms and without requiring any specific medical tests. Table 10 shows a comparison of the proposed model with various other models present in the literature. While prediction accuracy provides a cut-point performance measure, the AUC provides a holistic evaluation of the model’s classification performance. It is important to note that, for the current study, we consider only symptomatic attributes and routine clinical tests as the attributes for model design; however, it outperforms other existing models in the literature.

The proposed WaOEL model could be efficiently utilized to predict PCOS in clinical and healthcare environments by incorporating it into the patient care process. During initial consultations, patients generally submit information regarding basic symptoms, usually via digital forms connected to the hospital’s electronic health records (EHR). The model would analyze this information and produce a primary diagnosis for PCOS, which would help clinicians to evaluate whether further invasive test procedures, such as hormonal level tests or an ultrasound, would be necessary or not. Model risk prediction, along with clinicians’ perspectives, would further improve decision making, facilitating prompt diagnosis, detection of comorbidities, and customized treatment strategies. The symptomatic PCOS detection model proposed in this work, through deployment in public health initiatives or mobile applications for proactive screening in underprivileged regions, could be very beneficial for women; furthermore, the model could be improved by incorporating real-time learning capability from additional data sample inputs through incremental learning. The designed expert ML model could even help women in self-analysis for susceptibility to PCOS based on initial symptoms and fundamental health indicators.

## 6. Conclusions

Our primary objective in this research was to design and implement optimized ML models that assist in early symptomatic PCOS diagnosis. Since no study has been conducted in this direction to diagnose PCOS from the basic symptoms and biomarkers, a new dataset was implemented, with 12 relevant features, using the data blending technique. In this article, we propose ensembled models, designed by stacking seven base classifiers, and the DL model as a meta-classifier for early symptom-based prediction of PCOS. The meta-heuristic WaO algorithm determined the optimal combination of hyperparameters for the proposed EL model. The results obtained showed the superior performance of the WaOEL model, with the highest accuracy, 92.8%, and an AUC of 0.93, compared to RSOEL and CSOEL. It can be suggested from this study that the incorporation of stacking techniques and optimization results in a superior WaOEL model that can precisely predict PCOS without involving any expensive, painful, or invasive tests. Furthermore, a feature importance analysis was conducted using the SHAP framework to provide clinical insights for symptomatic PCOS predictions from the designed models. The analysis of the high-performing ensembled RF model suggested that attributes such as weight gain and BMI are key parameters leading to positive PCOS prediction; moreover, SHAP value analysis for XGB suggested that features including excessive hair growth and cycle length are also crucial. Findings using the proposed models support the clinical fact that patients with obesity and higher cholesterol are more likely to be diagnosed with PCOS.

Early symptomatic PCOS detection with the model will be helpful in an interdisciplinary clinical setup, resulting in better treatment planning and management, as well as avoiding further escalation to other lifestyle disorders. This model would reduce the financial burden on patients by decreasing the number of expensive clinical tests required for a PCOS diagnosis. The proposed framework does have certain limitations, such as increased computational complexity; therefore, the execution time required is greater, an issue that could be improved. Furthermore, future work in this direction could include the development of a mobile application framework with a more exhaustive and high-quality dataset and a larger number of data samples to test the robustness of the optimized model in real time.

## Figures and Tables

**Table 1:** Recent studies in the field of AI for disease diagnostics.

Reference	Applied Approach	Inference Made
Shamik et al. [42]	PCOS classification was analyzed by using classifiers such as SVM, DT, RF, LR, QDA, LDA, KNN, Gradient Boost, AdaBoost, extreme gradient boosting (XGB), and CatBoost.	By assessing accuracy using out-of-bag error analysis, RF showed better performance in PCOS diagnosis.
Elmannai et al. [43]	Employed basic ML models and AdaBoost. Hyperparameter optimization was used to determine the ideal hyperparameter.	Stacking ML with REF feature selection recorded the highest performance.
Prajna et al. [44]	Various models were employed, such as Chi-Square, DT, RF, and LR.	Results were generated by chatbots based on the parameters produced by users. However, chatbots can predict the possibility of PCOS but cannot provide a definite diagnosis.
Khanna et al. [45]	Feature selection was performed by applying the Harris Hawks Optimization algorithm. To improve performance, DL and XAI techniques were implemented.	The creation of an interface implemented real-time PCOS screening.
Shazia et al. [46]	The ten hyper-parametrized ML models were applied with the Gaussian Naive Bayes classifier.	Overfitting was seen in the models.
Vaidehi et al. [47]	The chi-square method was employed for feature selection, employing models such as RF, SVM, LR, GNB, and KNN.	The RF Classifier was the most accurate and dependable.
Ahmed et al. [48]	PCOS was detected using various ML techniques, including convolutional neural networks and the naive Bayes technique.	Results in an imbalanced dataset, decreased detection rate, noise in ultrasound images, and less use of clustering approaches.
Jyoti et al. [49]	Blood glucose levels were computed using support vector regression and extreme gradient boost regression algorithms.	The research led to the creation of a blood glucose surveillance device that uses a wristband and a mix of physiological and MFCC features to accurately predict blood glucose levels.

**Table 2:** A subset of the blended symptomatic dataset.

cp	trestbps	chol	fbs	restecg	thalach	Glucose	BMI	Cycle Length	Waist: Hip Ratio	Weight Gain (Y/N)	Hair Growth (Y/N)	PCOS (Y/N)
0	125	212	0	1	168	89	23.3	5	0.83	0	0	0
0	140	203	1	0	155	95	24.9	5	0.84	0	0	0
0	145	174	1	1	125	136	28.3	5	0.9	0	0	1
2	140	185	0	0	155	75	32	2	0.89	1	1	1
0	104	208	0	0	148	109	21.6	5	0.85	0	0	0
2	129	196	0	1	163	144	31.2	7	0.95	0	1	1
2	128	229	0	1	150	151	29.2	5	0.84	0	1	1
2	120	258	0	0	147	93	18.2	5	0.82	0	0	0
0	140	226	0	1	178	89	29.7	3	0.84	1	1	1
1	135	203	0	1	132	142	29.4	3	0.83	0	0	0

**Table 3:** Governing parameters for the metaheuristic algorithms.

Parameters of WaO	Utilized Value	Parameters of CSO	Utilized Value
Maximum number of iterations, *T*	50	Maximum number of iterations	50
Number of walrus/population, *N*	25	Number of nests/population	25
Number of decision variables, *m*	25	Number of optimizable variables	25
		The discovery rate of alien eggs	0.25

**Table 4:** Mean 10-fold cross-validation accuracy for base models.

Learning Algorithm	Mean Validation Accuracy (%)
KNN	79.3
SVM	80.5
LR	80.8
DT	83.2
RF	84.1
XGB	83.7
DL	81.0

**Table 5:** Performance measure of base learning models with testing data.

Model/ Metric	KNN	SVM	LR	DT	RF	XGB	DL
Accuracy	84.4	82.2	84.4	84.7	86.8	86.2	85.0
Precision	84.1	76.0	85.7	85.4	84.6	84.3	80.4
Sensitivity	63.8	65.5	62.1	70.7	75.9	74.1	70.7
Specificity	94.3	90.2	95.1	94.3	93.4	93.4	91.8
NPV	84.6	84.6	84.1	87.1	89.1	88.4	86.8
F1 score	72.6	70.4	72.0	77.4	80.0	78.9	75.2
FPR	05.7	09.8	04.9	05.7	06.6	06.6	08.2
FNR	36.2	34.5	37.9	29.3	24.1	25.9	29.3

**Table 6:** Performance measures of RSO-tuned EL models with various meta-classifiers.

Model/ Metric	KNN	SVM	DT	LR	RF	XG	DL
Accuracy	88.3	86.7	87.2	86.1	87.2	88.3	89.9
Precision	91.0	83.3	80.7	81.6	81.8	91.1	89.6
Sensitivity	69.0	71.4	79.3	71.4	77.6	70.7	74.1
Specificity	97.5	93.6	91.0	92.7	91.8	96.7	95.9
NPV	86.9	87.9	90.2	87.8	89.6	87.4	88.6
F1 score	79.2	76.9	80.0	76.2	79.7	79.6	81.1
FPR	02.5	06.5	09.0	07.3	08.2	03.3	04.1
FNR	31.0	28.6	20.7	28.6	22.4	29.3	25.9

**Table 7:** The hyperparameters considered for optimization with search space.

Algorithm	Hyperparameter Description	Search Space	Optimum Values for		
			RSOEL	CSOEL	WaOEL
LR	The inverse of regularization strength	[0.001, 100]	0.7	0.3	0.17
KNN	Number of neighbors	[8, 20]	13	8	10
SVM	The inverse of regularization strength	[0.001, 100]	0.04	0.21	0.063
DT	Min_samples_split	[2, 30]	5	2	29
	Min_samples_leaf	[2, 8]	7	2	13
	Max_leaf_nodes	[2, 20]	8	8	10
	Max_depth	[2, 20]	6	2	12
RF	n_estimators	[20, 150]	46	20	31
	max_depth	[2, 20]	9	16	6
	min_sample_split	[2, 8]	4	8	6
	min_sample_leaf	[2, 8]	2	2	2
	random_state	[0, 100]	61	61	94
XGB	learning_rate	[0.001, 0.5]	0.012	0.107	0.186
	max_depth	[2, 20]	11	19	15
	n_estimators	[10, 160]	25	54	72
DL	Hidden_layer_sizes (layer 1)	[4, 24]	6	22	23
	Hidden_layer_sizes (layer 2)	[10, 32]	15	10	14
	Hidden_layer_sizes (layer 3)	[20, 80]	48	24	70
	Hidden_layer_sizes (layer 4)	[4, 32]	30	10	18
	learning_rate	[0.001, 0.5]	0.02	0.018	0.022
Meta-Classifier DL	Hidden_layer_sizes (layer 1)	[4, 32]	10	8	8
	Hidden_layer_sizes (layer 2)	[8, 40]	11	18	18
	Hidden_layer_sizes (layer 3)	[20, 80]	56	46	42
	Hidden_layer_sizes (layer 4)	[4, 32]	30	16	18
	learning_rate	[0.001, 0.5]	0.007	0.011	0.14

**Table 8:** Average 10-fold cross-validation accuracy for optimized EL models.

Learning Model	Average Validation Accuracy (%)
RSOEL	86.3
CSOEL	88.6
WaOEL	90.1

**Table 9:** The performance measures for EL models with different optimizations.

Model	RSOEL	CSOEL	WaOEL
Accuracy	89.9	91.7	92.8
Precision	89.6	91.6	92.7
Sensitivity	74.1	91.7	92.8
Specificity	95.9	91.7	92.8
NPV	88.6	91.7	92.8
F1 score	81.1	91.6	92.7
FPR	04.1	8.3	7.2
FNR	25.9	8.3	7.2

**Table 10:** Comparative analysis with the existing literature.

Literature	Methods	Accuracy (%)	AUC
Tiwari et al. [42]	RF classifier with out-of-bag error tuning	92.4	0.91
Thakre et al. [47]	RF, SVM, LR, Gaussian Naïve Bayes, KNN	86.3	0.89
Zigrelli et al. [55]	RF classifier	82.5 (invasive tests), 90.0 (non-invasive parameters)	-
Proposed Technique	WaO tuned EL model	92.7	0.93

## Data Availability

CVD Dataset, available from: https://www.kaggle.com/datasets/johnsmith88/heart-disease-dataset (accessed on 10 June 2024). Polycystic ovary syndrome (PCOS), available from: https://www.kaggle.com/datasets/prasoonkottarathil/polycystic-ovary-syndrome-pcos (accessed on 22 June 2024).
